# A System of Dental Surgery

**Published:** 1873-07

**Authors:** 


### BIBLIOGRAPHICAL.

*A System of Dental Surgery.*—By John Tomes, F. R. S., and Charles S. Tomes, M. A. Second edition, revised and enlarged. Publishers, Lindsay & Blakiston, Philadelphia.

It is with pleasure that we announce the second edition of a work which nearly fifteen years ago was presented to the profession by an English author, who has acquired a world-wide reputation for his scientific researches, and his ability as a leading member of our profession in Europe.

While the text of this second edition has been considerably increased, the bulk of the volume has been lessened, owing to smaller type being used. Some sixty new illustrations have been added to the present edition, making in all the number of 263, and 748 pages. The chapters devoted to neuralgia, dentigerous cysts, odontomes, secondary affections resulting from the irritation set up by dental disease, and other shorter sections are entirely new. The chapter on dental caries, however, with the exception of slight additions and alterations remains much the same as in the first edition, but an appendix is added which gives a summary of the conclusions which the author draws from recent investigations.

Acknowledgment is made of the assistance derived from Heath's "Diseases and Injuries of the Jaws," Wedl's "Pathology of the Teeth," and the "Atlas" of Profs. Heider and Wedl.

As regards the theory of enamel formation, and causes of dental caries, the author's opinions conflict with those generally accepted in this country. We recommend the work as a useful addition to dental literature.

